# Linguistic-cognitive intervention in a case of early-onset Alzheimer’s disease

**DOI:** 10.1590/2317-1782/e20250126en

**Published:** 2026-06-01

**Authors:** Beatriz Andrade de Souza, Maria Isabel D’Ávila Freitas

**Affiliations:** 1 Universidade Federal de Santa Catarina – UFSC - Florianópolis (SC), Brasil.; 2 Departamento de Fonoaudiologia, Centro de Ciências da Saúde, Universidade Federal de Santa Catarina – UFSC - Florianópolis (SC), Brasil.

**Keywords:** Dementia, Early-Onset Alzheimer’s Disease, Cognitive Dysfunction, Language and Speech Disorder Rehabilitation, Speech-Language Pathology

## Abstract

Early-Onset Alzheimer’s Disease (EOAD) is an atypical variant of dementia that usually presents with impairments in language, visuospatial abilities, executive functions, or complex motor skills, with an early onset before the age of 65. This case study investigates the effect of a weekly language-cognitive intervention program in a patient with EOAD, diagnosed at the age of 58. The following protocols were applied for assessment before, during, and after the intervention: the Addenbrooke’s Cognitive Examination – Revised (ACE-R), the Arizona Battery for Communication Disorders of Dementia (ABCD), and the Boston Naming Test (BNT). This case study demonstrated that the language-cognitive intervention had a beneficial effect in maintaining linguistic expression abilities, despite the expected progression of a neurodegenerative disease.

## INTRODUCTION

Early-onset Alzheimer's Disease (AD) is an atypical variant of dementia usually manifested with changes in language, visuospatial skills, executive or complex motor functions, which start early, before the age of 65^(1)^. The less frequent clinical variants of AD are the logopenic variant of Primary Progressive Aphasia (lvPPA), Posterior Cortical Atrophy (PCA), also called the posterior variant, the Behavioral and Dysexecutive variant (bvFTD), and the Corticobasal Syndrome (CBS)^(1)^.

Unlike the amnestic variant of AD, in which the first symptom usually involves memory-related complaints, the less frequent variants have other early symptoms. PCA, for example, can initially cause difficulties in finding objects even when they are visibly accessible; difficulty with locomotion in irregular places, such as going up and down stairs; loss of manual dexterity; difficulty in gathering objects or getting dressed^(2)^. These difficulties are alterations more directly related to visuospatial impairments, which can also, secondarily, interfere with language.

Dementia is treated with pharmacological and non-pharmacological therapies, and an appropriate combination of both produces the best results^(3)^, with techniques and approaches that contribute to alleviating symptoms, delaying disease progression, and improving quality of life^(4)^. Speech-language intervention is an alternative to compensate for linguistic deterioration^(5)^. Given the above, the present case study aims to investigate the effect of a linguistic-cognitive intervention program on a patient with early-onset AD.

## CLINICAL CASE PRESENTATION

This study was approved by the Research Ethics Committee (REC) under opinion number 7.110.856. Information about the study was provided to the person responsible for the patient, who signed the Term of Free and Informed Consent (TFIC).

It is a female participant, 58 years old, with 24 years of schooling (doctorate completed), and a retired speech therapist. The patient had a medical diagnosis of early-onset AD in 2022, with the first symptoms in 2019, which were memory and organization difficulties related to the study and development of the doctoral thesis she was writing. The report of the magnetic resonance imaging of the skull, performed in 2022, indicated diffuse brain volume reduction with a slight parietal predominance, and the radiologist suggested possible posterior cortical atrophy, a diagnosis confirmed by the clinical neurologist, without an expanded neuropsychological evaluation at the time.

The patient began speech therapy in July 2023 at a speech therapy outpatient clinic specializing in acquired language and speech disorders at a university hospital. At this moment, the patient was using the anticholinesterase drug Rivastigmine for AD, but a family member reported that he did not notice improvement with its use. In the first interview, the patient reported difficulties in instrumental activities of daily living (IADL) and basic activities (ADL), such as dressing, for which she needed help. The Pfeffer Functional Activities Questionnaire indicated functional impairment compatible with dementia (score 15; scores greater than or equal to 5 are already indicative), with difficulties with tasks such as shopping, preparing meals, and managing finances.

For the linguistic-cognitive evaluation, the protocols “Addenbrooke's Cognitive Examination - Revised Version” were used for cognitive screening; “Arizona Battery for Communication Disorders in Dementia (ABCD)” for the evaluation of language and cognitive functions supporting language; and the “Boston Naming Test - BNT” to evaluate the ability to name figures.

The speech-language intervention was designed based on the combined cognitive intervention carried out in a previous study^(3)^, focusing on semantic lexical processing and using mixed therapeutic strategies of spaced retrieval and error-free learning based on reality orientation therapy. The main therapeutic objective was to maintain the patient's baseline communication and cognitive-linguistic skills. Moreover, exercises involving reading and writing tasks were associated to reinforce language stimulation.

The linguistic-cognitive training took place weekly from August 2023 to September 2024, with 50-minute sessions each. The intervention period had 28 sessions of speech therapy. Throughout the therapeutic process, the patient was reassessed approximately every 4 months of intervention, totaling 4 evaluation moments.

The intervention program was structured into therapeutic focuses, each subdivided into themes for each session (access the full document here: Google)^(6)^. The chosen subjects were designed according to the patient's reality, routine, functionality, and disease stage. Written tasks to be performed at home during the week were delivered to the patient to stimulate written language as a support for the linguistic-cognitive deficits observed in oral language.

## DISCUSSION

The main objective of this study was to investigate the effect of a linguistic-cognitive intervention program on a patient with early-onset AD.

Since the beginning of the intervention, the patient presented episodic memory deficit, disorientation in time and space, and ideomotor and constructional apraxia, compatible with PCA, which is one of the non-amnesic variants of AD^(2)^. The posterior variant of AD manifests itself between 50 and 60 years of age and is characterized by marked deficits in visuospatial perception and construction that interfere with other cognitive domains. These deficits can result, for example, in alexia, visual neglect syndrome, and limb apraxia^(2)^. Although in the early stages, episodic memory, language, and executive functions are still relatively preserved, the evolution of PCA leads to a more diffuse pattern of cognitive dysfunction^(7)^.

The patient presented language alterations, confirming what several studies indicate: that all other variants present language difficulties^(7-9)^. As for oral expression, the patient could utter grammatically correct sentences but had limitations in starting and maintaining a conversation, presenting evident lexical-semantic deficits, reflected in frequent pauses, anomalies, paraphasias, and repetitions during oral discourse. In PCA, when language alterations occur, they resemble a logopenic syndrome, with a predominance of anomalies, disfluencies, and impaired sentence repetition^(2)^. As a result of the impairment of memory and executive functions, the patient also presented communicative difficulties for shift changes, tangentiality of the topic during the conversation, difficulty in adapting her speech to the context quickly, as a spontaneous conversation requires, and in some moments, moderate deficits in understanding semi-complex and complex sentences were also observed.

Attentional and visuospatial difficulties were also present from the beginning of the intervention, which affected the performance of activities during therapy^(10)^, especially those involving written language, which was initially preserved but showed some flaws in graphomotor aspects and the presence of phonemic and morphemic paragraphs. It evolved with difficulty in forming semi-complex and complex sentences, writing only with loose words, perseveration of words, until she lost the ability to trace letters and the functionality of writing.

Each speech therapy session followed the same organization. Initially, there was a conversation with the patient about her week, and subsequently, her homework was analyzed with her, which included a board for writing the daily routine during the week and semantic and speech script tasks.

Afterwards, she was presented with the session topic and the words written on paper to be trained using the spaced retrieval technique. These words were directly related to the session theme. Visuospatial changes interfered with strategy execution but were reinforced through auditory and written channels, using an error-free learning technique.

The final part of the session focused on Reality Orientation Therapy (ROT), in which a printed calendar was presented to stimulate the patient's temporal orientation, followed by the theme for that session. Throughout the last activity, the patient was occasionally asked to recall the trained words.

During the ROT, the patient exhibited visuospatial difficulties in locating calendar information (year, month, day of the week, day of the month) and required assistance in all sessions. In some sessions, in the final part of the treatment, even with visual support from the calendar in front of her, she found it difficult to locate the requested items. Likewise, she had severe difficulty in memorizing such information.

In the spaced-word retrieval activity, the patient was rarely able to recall the words presented at the beginning of the session and was trained over increasing intervals throughout the session. Although the words were related to the chosen theme of the day, it was constantly necessary to provide semantic and phonological clues. Initially, the spaced retrieval technique was performed with five words, but due to the difficulty of evoking the patient, it was reduced to three words. As the disease progressed, the activity was performed in the last sessions with only one word.

In activities involving reading and writing, initially, there was no need for assistance. Probably due to the patient's high level of education, these skills were preserved^(11)^. However, gradually, visuospatial changes were observed, such as literal paragraphs, lack of self-correction, and writing outside the limits of the line, evolving into a degradation of the graphomotor trait to the point that much of the writing became intelligible, characteristic of a severe condition of profound dysgraphia due to neurodegenerative disease. Reading remained preserved for longer, but visuospatial and attentional failures compromised this ability, causing symptoms of mixed acquired dyslexia with literal paralexia that is observed in patients with dementia. Therefore, the weekly homework activities were suspended in the last months of the intervention due to a lack of functionality in reading and writing skills. Patients with AD with a predominance of posterior atrophy often have difficulty writing in a straight line, and reading ability may decrease due to poor spatial tracking ^(2)^.

Throughout the therapeutic process, the patient presented calm and collaborative behavior, but with reduced communicative initiative and difficulty in maintaining attention and eye contact during the dialogue. Whenever asked about her week, the answer was practically the same in all sessions: she reported, *"I cleaned the house, I had friends over to visit me"*.

Regarding the use of personal items such as glasses, sunglasses, and clothing, it was common in reading moments, for example, to have difficulty identifying which of the glasses to use (when she came with both), or even finding them, even though the item was hanging from a cord around her neck. Regarding clothing, in some sessions, the patient arrived with an inside-out blouse, a blouse worn the wrong way, or an untied shoelace.

Another point to be highlighted is the use of medication. In May 2024, the patient's husband (legal guardian) informed that he had interrupted the pharmacological treatment, as the patient was presenting gastrointestinal symptoms and he did not perceive positive effects of the drug on the course of the disease. Even knowing the importance of the anticholinesterase drug for AD, and being instructed to continue with the treatment, he kept only the use of Memantine that the patient was using at that moment.

Regarding self-perception of speech therapy, when asked about changes in communication after she started speech therapy, she reported improvement: "Before I was afraid to speak because I was stuck, now, even with difficulties, I do not stop expressing myself." This report shows a direct impact on their well-being and quality of life, aspects highlighted by intervention studies with those patients^(3,5,8)^.

In the last months of the intervention, the degradation of visuospatial skills began to directly impact the planning and execution of activities. The patient started to have intense symptoms of visual neglect, not recognizing objects and figures in front of her, even when receiving help.

To better interpret the effects of the speech therapy intervention and the evolution of the disease, a descriptive analysis of the four evaluation moments was initially conducted. Regarding the results obtained in the application of the *Addenbrooke’s Cognitive Examination –* Revised (ACE-R), we note that over these 12 months of follow-up, there was a significant reduction in the patient's cognitive performance (total ACE-R value across the four evaluations: 53, 50, 44, 30), particularly in the last evaluation conducted in September 2024. When we analyze each evaluated construct individually, we can highlight a greater drop in the "Attention and Orientation" tests (performance in the four evaluations: 8, 9, 6, and 3), which corroborates the cognitive profile of the AD variant that the patient presents^(2)^.

In the Arizona Battery for Communication Disorders in Dementia (ABCD), through the score of each construct throughout the evaluations, we can notice a sharper drop in the patient's performance in the constructs of “Linguistic Comprehension” (performance in the four evaluations: 63, 37, 22, and 36) and “Visuospatial Construction” (performance in the four evaluations: 10, 12, 13, and 5). As previously mentioned, language deficits are observed in AD patients, and comprehension failures may occur due to visuospatial dysfunction that extends to other cognitive domains such as memory, attention, and reasoning^(7)^. In the Boston Naming Test (BNT), the number of correct answers without a clue over time did not vary greatly (performance in the four evaluations: 39, 33, 40, and 33), which may be attributed to the patient's cognitive reserve, a predictor of better performance in linguistic functions^(11)^.

For the inferential statistical analysis of the patient's performance, a linear regression was also performed, which involves plotting a line between a dependent and an independent variable to explain their relationship. In this case study, each evaluation protocol was treated as a dependent variable as a function of time, with time as the independent factor, since we have four distinct periods of patient evaluation.

In this way, it is possible to evaluate the slope of the curve, that is, the slope of the line. Whenever the slope is non-zero, it will have a positive or negative value. Since the slope to the positive indicates an increase in the parameters, the slope to the negative indicates a reduction. In this case, the parameters are the evaluation protocols and their respective subtests. To determine whether the slope of the curve was statistically significant, the p-values of the parameters were calculated in view of the adopted significance level. The statistical significance level used was 0.05 (5%).

**Figure 1:**
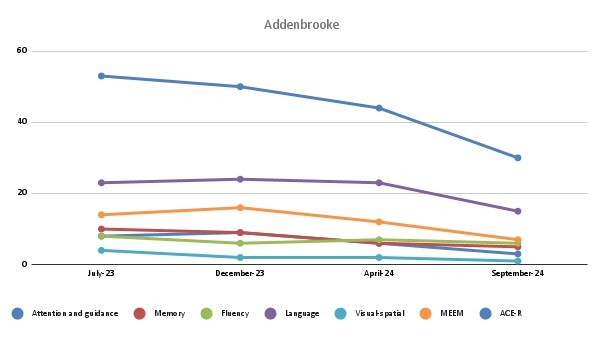
Slope of the line for each subtest and the total score of the Addenbrooke’s Cognitive Examination – Revised (ACE-R)

Among the three evaluation protocols, we observe a greater drop in ACE-R scores than in the other tests. The slope of the line in this protocol and its respective subtests is best exemplified in Figure 1. We can justify these findings due to the fact that deficits in the domains of attention, reasoning and memory are influenced by visuospatial impairment, which is the most affected in the patient's AD variant ^(2)^. The other two instruments (ABCD and BNT) assess language skills more specifically.

The results showed a statistically significant reduction only in the patient's performance on the Memory construct and in the total ACE-R score (Table 1). It is widely recognized that the most common initial manifestation in patients with late-onset AD is memory deficit. In cases of early-onset AD, this memory impairment will occur as the disease progresses, as well as deficits in language and other cognitive functions^(1)^.

**Table 1:** Slope of the longitudinal curve of the analyzed parameters and their respective p-values

**Evaluation Protocol**		**Slope**	**p-value**
*Addenbrooke (ACE-R)*	Attention and orientation	-1.80	0.122
	Memory	-1.80	0.024
	Fluency	-0.50	0.326
	Language	-2,50	0.230
	Visuospatial	-0.90	0.077
	ACE-R Total Score	-7.50	0.052
*Arizona (ABCD)*	Mental state	- x -	- x -
	Episodic memory	1.10	0.673
	Linguistic expression	-3.10	0.364
	Linguistic understanding	-9.60	0.275
	Subtest- Reading comprehension (sentences)**	-1.90	0.019
	Visuospatial	-1.40	0.492
*Boston Naming Test (BNT)*		-1.10	0.624

** Reading comprehension (sentences) corresponds to one of the subtests of the linguistic comprehension construct

**Caption:** Slope = Slope of the curve, which can be equal to or different from zero, and the slope can be positive or negative. Whenever the slope is to the positive, it indicates an increase, while when the slope is negative, it indicates a reduction; p-value = How much the variation of the curve is statistically significant, given the significance level adopted; -x- = It was not possible to perform the statistical analysis

In the language tests, there was a statistically significant worsening only in the ABCD “Reading comprehension (sentence)” subtest. Table 1 also shows all slope and p-value results for ABCD and BNT. We believe that the significant reduction in only one subtest of the language battery (ABCD) is due to the combination of a memory deficit associated with visuospatial dysfunction, visual neglect syndrome, and impaired spatial tracking, generated by the posterior variant of AD^(2)^. On the other hand, we believe that the slighter drop in language expression skills may be a consequence of the language stimulation performed in speech therapy that may have preserved the patient's oral language to some extent^(12)^

It is noteworthy that since the beginning of consultations, the patient has had difficulty with visuospatial perception and construction, which has directly affected other cognitive domains, including memory, language, attention, and reasoning. Likewise, from the beginning of the intervention, she had an impoverished speech and mild comprehension failures that worsened at the end of the treatment. Because it is a neurodegenerative disease, a reduction in cognitive performance is expected^(9)^.

However, the overall maintenance of scores on the ABCD and BNT tests suggests that speech-language pathology intervention played a protective role in these skills. Cognitive stimulation is a non-pharmacological intervention that has been gaining popularity in recent years. Currently, some types of cognitive training have been validated for use^(3)^, among them are those used in our study. This preservation can also be attributed, as already mentioned, to the patient's cognitive reserve ^(11)^.

Thus, the combination of spaced evocation techniques, error-free learning, and reality orientation therapy proved effective in promoting communicative skills^(12)^.

As the patient's cognitive condition worsened throughout the intervention period, in the final months, we chose to focus exclusively on themes related to daily activities. Functionality is an aspect strongly affected in dementia^(4)^. An important point is that functional changes in cases of posterior variant AD can be directly influenced by visuospatial perception and construction deficits^(2)^.

The treatment of dementia involves both pharmacological and non-pharmacological therapies, and the proper integration of these approaches tends to yield the most effective results^(3)^. A review of published guidelines and recommendations on the discontinuation of the anticholinesterase drug^(13)^ revealed a lack of clinical trial data, making it difficult to build a solid evidence base for the practice. From common side effects such as gastrointestinal symptons, to serious adverse effects such as seizures, families often give up drug treatment^(13)^.

The progression of the disease in the final months and the significant worsening of visuospatial skills directly impacted the therapeutic strategies, which made it difficult to proceed with the sessions. As a consequence, a therapeutic limit was established, and the linguistic-cognitive intervention was terminated. The family was instructed to stimulate the patient daily with the activities of her routine. At this stage of the disease, group activities, music therapy, regular physical activity such as walking, active and functional participation in their routine, and stimulating visits from friends and family promote the patient's well-being and positively impact the quality of life^(14)^.

The findings of this study reinforce the importance of individualized interventions that consider the functional reality and specific needs of each patient^(8)^. The progression of cognitive decline, especially in visuospatial functions, showed the need to continuously adapt therapeutic strategies to maximize patient functionality and quality of life.

The limitations of this study include the temporary interruption of sessions during school holidays at the educational institution where the treatment was carried out and the possible interference of the suspension of anticholinesterase pharmacological treatment.

However, given the scarcity of studies on speech-language intervention for language stimulation in patients with early-onset AD, we believe the present case report can contribute to the development of scientific evidence in this area of speech-language pathology.

## FINAL COMMENTS

This case study demonstrated that a linguistic-cognitive intervention based on strategies such as spaced retrieval, error-free learning, and reality orientation therapy, integrated with reading and writing activities, had a beneficial effect on maintaining language expression skills in a patient with early-onset AD, despite the expected progression of a neurodegenerative disease.
