# Supplementary material for: Linguistic-cognitive intervention in a case of early-onset Alzheimer’s disease
Source: Codas. 2026 Jun 1;38(3):e20250126. doi: 10.1590/2317-1782/e20250126en (PMC13288443; doi:10.1590/2317-1782/e20250126en)
Supplement: Translation

# Translation: Intervenção linguístico-cognitiva em um caso de doença de Alzheimer de início precoce

**DOI:** 10.1590/2317-1782/e20250126pt

**Authors:** Beatriz Andrade de Souza, Maria Isabel D’Ávila Freitas

**Affiliations:** 1 Universidade Federal de Santa Catarina – UFSC - Florianópolis (SC), Brasil.; 2 Departamento de Fonoaudiologia, Centro de Ciências da Saúde, Universidade Federal de Santa Catarina – UFSC - Florianópolis (SC), Brasil.

**Keywords:** Demência, Doença de Alzheimer de Início Precoce, Disfunção Cognitiva, Reabilitação dos Transtornos da Linguagem e da Fala, Fonoaudiologia

## Abstract

A Doença de Alzheimer (DA) de Início Precoce é uma variante atípica de demência e costuma se manifestar com alterações da linguagem, das habilidades visuais-espaciais, das funções executivas ou motoras complexas, que tem início precoce, antes dos 65 anos. Esse estudo de caso investiga o efeito de um programa de intervenção linguístico-cognitiva realizado de forma semanal em uma paciente com DA de início precoce, diagnosticada aos 58 anos. Foram aplicados os protocolos Exame Cognitivo de Addenbrooke - Versão Revisada (ACE-R), Bateria Arizona para Desordens de Comunicação na Demência (ABCD) e Teste de Nomeação de Boston (BNT) para avaliação pré, durante e após intervenção. Este estudo de caso demonstrou que a intervenção linguístico-cognitiva teve efeito benéfico de manutenção das habilidades de expressão linguística, apesar da progressão esperada de uma doença neurodegenerativa.

## INTRODUÇÃO

A Doença de Alzheimer (DA) de Início Precoce é uma variante atípica de demência e costuma se manifestar com alterações da linguagem, das habilidades visuais-espaciais, das funções executivas ou motoras complexas, que tem início precoce, antes dos 65 anos^(1)^. As variantes clínicas da DA menos frequentes são a variante logopênica da Afasia Progressiva Primária (vlAPP), Atrofia Cortical Posterior (ACP) ou também chamada de variante posterior, a variante Comportamental e Disexecutiva (vcdDA) e a Síndrome corticobasal (SCB)^(1)^.

Diferente da variante amnésica da DA em que o primeiro sintoma geralmente envolve queixas relacionadas à memória, as variantes menos frequentes apresentam outros sintomas iniciais. A ACP, por exemplo, inicialmente pode ocasionar dificuldades para encontrar objetos mesmo quando estão visivelmente acessíveis; dificuldade para locomoção em lugares irregulares, como subir e descer escadas; perda da destreza manual; dificuldade para juntar objetos ou se vestir^(2)^. Essas dificuldades são alterações mais diretamente relacionadas a comprometimentos visuoespaciais, que também podem interferir, secundariamente, no domínio da linguagem.

A demência é tratada com terapias farmacológicas e não farmacológicas, e uma combinação apropriada de ambas produz os melhores resultados^(3)^, com técnicas e abordagens que contribuem para aliviar os sintomas, retardar a progressão da doença e melhorar a qualidade de vida^(4)^. A intervenção fonoaudiológica é uma alternativa para compensar a deterioração linguística^(5)^. Diante do exposto, o presente estudo de caso tem como objetivo investigar o efeito de um programa de intervenção linguístico-cognitiva em uma paciente com DA de início precoce.

## APRESENTAÇÃO DO CASO CLÍNICO

O presente estudo foi aprovado pelo Comitê de Ética em Pesquisa (CEP) sob o parecer de número 7.110.856. As informações sobre o estudo foram prestadas ao responsável pela paciente, que assinou o Termo de Consentimento Livre e Esclarecido (TCLE).

Trata-se de uma participante do sexo feminino, 58 anos, 24 anos de escolaridade (doutorado concluído), fonoaudióloga aposentada. A paciente teve o diagnóstico médico de DA de início precoce em 2022, com os primeiros sintomas em 2019, sendo eles dificuldade de memória e organização relacionadas ao estudo e desenvolvimento da tese de doutorado que estava escrevendo. O laudo da ressonância magnética de crânio, realizado em 2022, indicou redução volumétrica encefálica difusa com leve predomínio parietal, sendo sugerido pelo médico radiologista uma possível atrofia cortical posterior, confirmada pelo médico neurologista clínico, sem ter sido submetida a uma avaliação neuropsicológica ampliada na ocasião.

A paciente iniciou acompanhamento fonoaudiológico em julho de 2023 em um ambulatório de Fonoaudiologia especializado em distúrbios neurológicos adquiridos de linguagem e fala de um hospital universitário. Neste momento, a paciente fazia uso de medicamento anticolinesterásico Rivastigmina para a DA, mas o familiar relatava que não percebia melhora com o uso. Na entrevista inicial, relatou dificuldades nas atividades instrumentais de vida diária (AIVD) e atividades básicas (AVD), como ao se vestir, necessitando ajuda. O Questionário de Atividades Funcionais de Pfeffer indicou prejuízo funcional compatível com demência (escore 15; valores maiores ou iguais a 5 já são indicativos), com dificuldades em tarefas como fazer compras, preparar refeições e controlar finanças.

Para avaliação linguístico-cognitiva foram utilizados os protocolos “Exame Cognitivo de Addenbrooke - Versão Revisada” para rastreio cognitivo; “Bateria Arizona para Desordens de Comunicação na Demência (ABCD)” para avaliação da linguagem e funções cognitivas de suporte da linguagem; “Teste de Nomeação de Boston - BNT” para avaliar habilidade de nomeação de figuras.

A intervenção fonoaudiológica foi delineada com base na intervenção cognitiva combinada realizada em estudo prévio^(3)^, com foco no processamento léxico semântico, no qual foram utilizadas estratégias terapêuticas mistas de evocação espaçada e aprendizagem sem erro baseadas na terapia de orientação para a realidade. O objetivo terapêutico principal foi a manutenção da comunicação e das habilidades cognitivo-linguísticas que a paciente apresentava na linha de base. Além disso, foram associados exercícios envolvendo tarefas de leitura e escrita para reforçar a estimulação da linguagem.

O treinamento linguístico-cognitivo aconteceu de agosto de 2023 a setembro de 2024, semanalmente, com sessões de 50 minutos cada. O período de intervenção contou com 28 sessões de terapia fonoaudiológica. Ao longo do processo terapêutico, a paciente foi reavaliada a cada 4 meses de intervenção aproximadamente, totalizando quatro momentos diferentes de avaliação.

A estrutura do programa de intervenção foi dividida em focos terapêuticos subdivididos em temas para cada sessão (acesse o documento completo aqui: Google)^(6)^. Os assuntos escolhidos foram pensados de acordo com a realidade, rotina, funcionalidade e estágio da doença da paciente. Tarefas escritas para serem realizadas em casa durante a semana eram entregues à paciente, com o objetivo de estimular a linguagem escrita como um suporte para os déficits linguístico-cognitivos observados na linguagem oral.

## DISCUSSÃO

O objetivo principal deste estudo foi investigar o efeito de um programa de intervenção linguístico-cognitiva em uma paciente com DA de início precoce.

Desde o início da intervenção, a paciente apresentava déficit de memória episódica, desorientação no tempo e no espaço, apraxia ideomotora e constructional compatível com a ACP, que é uma das variantes não amnésicas da DA^(2)^. A variante posterior da DA manifesta-se entre 50 e 60 anos e caracteriza-se por acentuados déficits na percepção e construção visuoespacial que interfere nos demais domínios cognitivos. Esses déficits podem resultar, por exemplo, em alexia, síndrome de negligência visual e apraxia de membros^(2)^. Embora nas fases iniciais a memória episódica, linguagem e funções executivas ainda estejam relativamente preservadas, a evolução da ACP leva a um padrão mais difuso de disfunção cognitiva^(7)^.

A paciente apresentava alterações de linguagem, confirmando o que diversos estudos apontam, que todas as outras variantes cursam com dificuldades linguísticas^(7-9)^. Quanto à expressão oral, a paciente conseguia elaborar frases gramaticalmente corretas, porém com limitações para iniciar e manter uma conversa, apresentando déficits léxico-semânticos evidentes, que se refletiam na comunicação da paciente com pausas frequentes, anomias, parafasias e paráfrases durante o discurso oral. Na ACP, quando as alterações de linguagem surgem são semelhantes a uma síndrome logopênica, predominando anomias, disfluências e repetição de frases prejudicadas^(2)^. Em decorrência do comprometimento da memória e funções executivas, a paciente apresentava também dificuldades comunicativas para trocas de turno, tangencialidade do tópico durante a conversa, dificuldade para adaptar seu discurso ao contexto de forma rápida como exige uma conversa espontânea, e em alguns momentos, déficits moderados de compreensão de frases semi-complexas e complexas também eram observados.

Dificuldades atencionais e visuoespaciais também estavam presentes desde o início da intervenção, o que afetava a realização das atividades durante a terapia^(10)^, especialmente naquelas que envolviam a linguagem escrita, que inicialmente era preservada mas com algumas falhas no aspecto grafomotor e presença de paragrafias fonêmicas e morfêmica. Evoluiu com dificuldade em formar frases semi complexas e complexas, escrita apenas de palavras soltas, perseveração de palavras, até perder o traçado da letra e a funcionalidade da escrita.

Cada sessão de terapia fonoaudiológica seguiu a mesma organização. Inicialmente, era feita uma conversa com a paciente sobre como foi a sua semana e, na sequência, analisava-se com ela a tarefa enviada para casa, que era composta de um quadro para escrever a rotina diária da semana e tarefas semânticas e de script de fala.

Após, introduzia-se o tópico da sessão e, em seguida, eram apresentadas as palavras escritas em um papel a serem treinadas com o uso da técnica de evocação espaçada, que possuiam relação direta com o tema da sessão. Alterações visuoespaciais interferiram na execução da estratégia, porém eram reforçadas através da entrada auditiva e escrita com base na técnica de Aprendizagem sem erro.

A parte final da sessão era destinada a Terapia de Orientação para a Realidade (ROT), onde se apresentava o calendário impresso para estimular a orientação temporal da paciente seguido do tema escolhido para conversar naquela sessão. Ao longo dessa última atividade havia momentos em que era solicitado que a paciente lembrasse das palavras treinadas.

Durante a ROT, a paciente apresentava dificuldade visuoespacial para conseguir localizar as informações do calendário (ano, mês, dia da semana, dia do mês) e precisou de auxílio em todas as sessões. Em algumas sessões, na parte final do tratamento, até com apoio visual do calendário a sua frente era difícil para ela conseguir localizar os itens solicitados. Da mesma forma, havia dificuldade grave para memorizar tais informações.

Na atividade de evocação espaçada de palavras, eram raras as vezes em que a paciente conseguia recordar as palavras apresentadas no início da sessão e treinadas em espaços de tempo crescentes ao longo da sessão. Apesar das palavras terem relação com o tema escolhido do dia, fazia-se necessário de forma constante fornecer pistas semânticas e fonológicas. Inicialmente, a técnica de evocação espaçada foi realizada com cinco palavras, mas devido à dificuldade de evocação da paciente reduziu-se para três palavras. Com o avanço da doença, a atividade foi realizada nas últimas sessões com apenas uma palavra.

Nas atividades que envolviam habilidade de leitura e escrita, inicialmente, não havia necessidade de auxílio. Provavelmente, pelo alto nível de escolaridade da paciente, havia a preservação dessas habilidades^(11)^. Porém, aos poucos, alterações visuoespaciais foram sendo observadas, como paragrafias literais, falta de autocorreção, escrita fora dos limites da linha, evoluindo para degradação do traço grafomotor ao ponto de grande parte da escrita se tornar inteligível, característica de um quadro grave de disgrafia profunda pela doença neurodegenerativa. A leitura manteve-se preservada por mais tempo, mas falhas visuoespaciais e atencionais foram comprometendo essa habilidade, causando um quadro de dislexia adquirida mista com paralexia literal que se observa em pacientes com demência. Por isso, as atividades que eram enviadas para casa semanalmente foram suspensas nos meses finais da intervenção por falta de funcionalidade das habilidades de leitura e escrita. Pacientes com DA com predomínio de atrofia posterior costumam apresentar dificuldade para escrever em linha reta e a capacidade de leitura pode diminuir devido ao mau rastreamento espacial^(2)^.

Ao longo de todo processo terapêutico, a paciente apresentou comportamento calmo e colaborativo, mas com iniciativa comunicativa reduzida com dificuldade na manutenção da atenção e do contato visual durante o diálogo. Sempre que questionada sobre como foi a sua semana, a resposta era praticamente a mesma em todas as sessões, relatava *“arrumei a casa, recebi alguns amigos que vieram me visitar”*.

Com relação ao uso de itens pessoais como óculos de grau, óculos de sol e vestimentas, era comum nos momentos de leitura por exemplo, apresentar dificuldade para identificar qual dos óculos utilizar (quando vinha com os dois), ou até mesmo tinha dificuldade para encontrá-lo, mesmo o item estando pendurado em um cordão no seu pescoço. Com relação à vestimenta, durante o tratamento houve sessões em que a paciente chegou até o atendimento com a blusa do avesso, blusa vestida de forma errada e cadarço desamarrado.

Outro ponto a ser destacado é o uso da medicação. Em maio de 2024, o marido da paciente (responsável legal) informou que havia interrompido o tratamento farmacológico com anticolinesterásico, pois a paciente estava apresentando sintomas gastrointestinais e ele não percebia efeitos positivos do medicamento no curso da doença. Mesmo sabendo da importância do medicamento anticolinesterásico para a DA, e sendo orientado a continuar com o tratamento, ele manteve apenas o uso da Memantina que a paciente estava em uso naquele momento.

Com relação à autopercepção sobre o tratamento fonoaudiológico, sempre que a paciente era questionada sobre mudanças para se comunicar depois que iniciou o acompanhamento fonoaudiológico, a mesma relatava melhora na comunicação, *“antes eu tinha medo de falar por travar, agora mesmo que com dificuldades eu não deixo de me expressar”*. Esse relato evidencia um impacto direto no seu bem estar e qualidade de vida, aspectos que são trazidos pelos estudos de intervenção com esses pacientes^(3,5,8)^.

Nos últimos meses da intervenção, a degradação das habilidades visuoespaciais passou a impactar de forma direta no planejamento e execução das atividades. A paciente passou a ter sintomas intensos de negligência visual, não reconhecendo objetos e figuras à sua frente, mesmo recebendo auxílio.

Para uma melhor interpretação dos efeitos da intervenção fonoaudiológica e da evolução da doença em si foi realizada inicialmente uma análise descritiva dos quatro momentos de avaliação. Com relação aos resultados obtidos na aplicação do *Exame Cognitivo de Addenbrooke -* Versão Revisada (ACE-R), podemos destacar que ao longo desses 12 meses de acompanhamento houve uma redução importante do desempenho cognitivo da paciente (valor total do ACE-R nas quatro avaliações: 53, 50, 44, 30), especialmente na última avaliação realizada em setembro de 2024. Quando analisamos de forma individual cada construto avaliado podemos destacar uma maior queda nas provas de “Atenção e Orientação” (desempenho nas quatro avaliações: 8, 9, 6 e 3), o que corrobora com o perfil cognitivo da variante da DA que a paciente apresenta^(2)^.

Na Bateria Arizona para Desordens de Comunicação na Demência (ABCD), através da pontuação de cada construto ao longo das avaliações podemos notar uma queda mais acentuada no desempenho da paciente nos construtos de “Compreensão linguística” (desempenho nas quatro avaliações: 63, 37, 22 e 36) e “Construção visuo-espacial” (desempenho nas quatro avaliações: 10, 12, 13 e 5). Como já mencionado anteriormente, déficits de linguagem são observados em pacientes com DA e falhas de compreensão podem ocorrer devido a disfunção visuoespacial atravessar outros domínios cognitivos como memória, atenção e raciocínio^(7)^. Já no Teste de Nomeação de Boston (BNT), o número de acertos sem pista ao longo do tempo não sofreu grandes variações, (desempenho no teste BNT nas quatro avaliações: 39, 33, 40 e 33) o que pode estar atribuído a reserva cognitiva da paciente, fator preditor de melhor desempenho em funções linguísticas^(11)^.

Para a análise estatística inferencial do desempenho da paciente foi realizada também uma análise de Regressão Linear, que basicamente consiste em traçar uma curva entre um fator dependente e outro independente, para que se possa explicar a relação entre eles. Nesse estudo de caso, cada protocolo de avaliação foi considerado como uma variável dependente em função do tempo, sendo o tempo o fator independente, já que temos quatro períodos diferentes de avaliação da paciente.

Desta forma, é possível avaliar o Slope da curva, ou seja, a inclinação da reta. Sempre que a inclinação for diferente de zero ela vai ter um valor positivo ou negativo. Sendo que a inclinação para o positivo indica aumento dos parâmetros, já a inclinação para o negativo indica redução. Nesse caso, os parâmetros são os protocolos de avaliação e seus respectivos subtestes. Para concluir se a inclinação da curva foi estatisticamente significante ou não perante o nível de significância adotado, foi realizado o cálculo do p-valor dos parâmetros. O nível de significância estatística utilizado foi de 0,05 (5%).

Dentre os três protocolos de avaliação utilizados, podemos perceber uma maior queda de pontuações do ACE-R do que nos demais testes. A inclinação da reta neste protocolo e seus respectivos subtestes está melhor exemplificada na Figura 1. Podemos justificar tais achados devido ao fato de déficits nos domínios de atenção, raciocínio e memória serem influenciados pelo prejuízo visuoespacial, que é o mais afetado na variante da DA da paciente^(2)^. Os outros dois instrumentos (ABCD e BNT) avaliam mais especificamente as habilidades de linguagem.

**Figura 1:** Inclinação da reta referente a cada subteste e ao valor total do Exame Cognitivo de Addenbrooke - Versão Revisada (ACE-R)

Os resultados mostraram que houve uma redução estatisticamente significativa apenas no desempenho da paciente no construto de Memória e na pontuação total do ACE-R (Tabela 1). É amplamente reconhecido que a manifestação inicial mais comum em pacientes com DA de início tardio é o déficit de memória. Já em casos de DA de início precoce, esse prejuízo de memória vai ocorrer com o avanço da doença, assim como, déficits de linguagem e outras funções cognitivas^(1)^.

**Tabela 1:** Slope de inclinação da curva longitudinal dos parâmetros analisados e seus respectivos P-valores

**Protocolo de Avaliação**		**Slope**	**P-valor**
*Addenbrooke (ACE-R)*	Atenção e orientação	-1,80	0,122
	Memória	-1,80	0,024
	Fluência	-0,50	0,326
	Linguagem	-2,50	0,230
	Visuoespacial	-0,90	0,077
	Pontuação Total do ACE-R	-7,50	0,052
*Arizona (ABCD)*	Estado mental	- x -	- x -
	Memória episódica	1,10	0,673
	Expressão linguística	-3,10	0,364
	Compreensão linguística	-9,60	0,275
	S*ubteste - Compreensão de leitura (sentenças)* ^**^	-1,90	0,019
	Visuoespacial	-1,40	0,492
*Teste de nomeação de Boston (BNT)*		-1,10	0,624

** Compreensão de leitura (sentenças) corresponde a um dos subtestes do construto de compreensão linguística

**Legenda:** Slope = Inclinação da curva, podendo ser igual ou diferente de zero, sendo que a inclinação pode ser para o positivo ou negativo. Sempre que a inclinação for para o positivo indica aumento, já quando a inclinação é negativa indica redução; P-valor = O quanto a variação da curva é estatisticamente significante, perante o nível de significância adotado; -x- = Não foi possível realizar a análise estatística

Nos testes de linguagem, houve piora estatisticamente significativa apenas no subteste de “Compreensão de leitura (sentença)” da ABCD. Todos os resultados de Slope e p-valor referente a ABCD e BNT podem ser consultados também na Tabela 1. Acreditamos que a redução significativa em apenas um subteste da bateria de linguagem (ABCD) seja devido à combinação de déficit de memória associado à disfunção visuoespacial, síndrome da negligência visual e do rastreamento espacial prejudicado, geradas pela variante posterior da DA^(2)^. Por outro lado, acreditamos que a queda menos acentuada nas habilidades de expressão linguística pode ser consequência da estimulação de linguagem realizada no tratamento fonoaudiológico que pode ter preservado em certa medida a linguagem oral da paciente^(12)^.

Vale ressaltar que a paciente desde o início dos atendimentos apresentou dificuldade na percepção e construção visuoespacial, impactando diretamente em outros domínios cognitivos como a memória, linguagem, atenção e o raciocínio. Da mesma forma, desde o início da intervenção tinha um discurso empobrecido e falhas de compreensão leve que se agravaram ao final do tratamento. Por se tratar de uma doença neurodegenerativa, é esperado que exista redução no desempenho cognitivo^(9)^.

Contudo, a manutenção geral da pontuação nos testes ABCD e BNT sugere que a intervenção fonoaudiológica desempenhou um papel protetor nessas habilidades. A estimulação cognitiva é uma forma de intervenção não farmacológica que vem ganhando força nos últimos anos. Atualmente, existem alguns tipos de treinamento cognitivos validados para uso^(3)^, dentre eles, os que foram utilizados no nosso estudo. Essa preservação também pode ser atribuída, como já referido, à reserva cognitiva da paciente^(11)^.

Dessa forma, a combinação das técnicas de evocação espaçada, aprendizagem sem erro e terapia de orientação para a realidade mostrou-se eficaz na promoção de habilidades comunicativas^(12)^.

Com a piora do quadro cognitivo da paciente ao longo do período de intervenção, nos meses finais optou-se por trabalhar com temas exclusivamente relacionados às atividades de vida diária. A funcionalidade é um aspecto fortemente afetado em quadros demenciais^(4)^. Uma informação importante é que as alterações funcionais nos casos de DA variante posterior podem sofrer influência direta dos déficits de percepção e construção visuoespacial^(2)^.

O tratamento da demência envolve tanto terapias farmacológicas quanto não farmacológicas, e a integração adequada dessas abordagens tende a oferecer os resultados mais eficazes^(3)^. Uma revisão das diretrizes e recomendações publicadas sobre a descontinuação do medicamento anticolinesterásico^(13)^ revelou uma escassez de dados provenientes de ensaios clínicos, o que dificulta a formação de uma base sólida de evidências para a prática. Desde efeitos colaterais comuns, como sintomas gastrointestinais até efeitos adversos graves como convulsões, fazem com que a família muitas vezes desista do tratamento medicamentoso^(13).^

A progressão da doença nos meses finais e a piora importante das habilidades visuoespaciais impactou diretamente na realização das estratégias terapêuticas, o que dificultou o andamento das sessões. Como consequência, foi estabelecido um limite terapêutico e optado por encerrar a intervenção linguístico-cognitiva. A família foi orientada a estimular diariamente a paciente com as atividades da sua rotina. Neste estágio da doença, atividades em grupo, musicoterapia, atividade física regular como caminhada, participação de forma ativa e funcional em sua rotina, estímulo à visita de amigos e familiares irão gerar bem-estar para a paciente impactando positivamente na qualidade de vida^(14)^.

Os achados deste estudo reforçam a importância de intervenções individualizadas que considerem a realidade funcional e as necessidades específicas de cada paciente^(8)^. A progressão do declínio cognitivo, especialmente nas funções visuoespaciais, mostrou a necessidade de adaptar continuamente as estratégias terapêuticas para maximizar a funcionalidade e a qualidade de vida do paciente.

São limitações deste estudo a interrupção temporária das sessões no período de férias escolares na instituição educacional onde o tratamento foi realizado, e a possível interferência da suspensão do tratamento farmacológico com o anticolinesterásico.

Contudo, dada a escassez de estudos sobre intervenção fonoaudiológica na estimulação da linguagem de pacientes com DA de início precoce, acreditamos que o presente relato de caso possa contribuir com a construção de evidências científicas nessa área de atuação fonoaudiológica.

## COMENTÁRIOS FINAIS

Este estudo de caso demonstrou que a intervenção linguístico-cognitiva, baseada em estratégias como a evocação espaçada, aprendizagem sem erro e a terapia de orientação para a realidade associada a atividades de leitura e escrita teve efeito benéfico de manutenção das habilidades de expressão linguística em uma paciente com DA de início precoce, apesar da progressão esperada de uma doença neurodegenerativa.
